# How Does Employee Green Behavior Impact Employee Well-Being? An Empirical Analysis

**DOI:** 10.3390/ijerph18041669

**Published:** 2021-02-09

**Authors:** Baojie Zhang, Lifeng Yang, Xiangyang Cheng, Feiyu Chen

**Affiliations:** 1 School of Business, Fuyang Normal University, Fuyang 236037, China; 19210501@stu.fynu.edu.cn (B.Z.); 198408002@fynu.edu.cn (X.C.); 2 School of Economics, Fuyang Normal University, Fuyang 236037, China; 3 School of Economics and Management, China University of Mining and Technology, Xuzhou 221116, China

**Keywords:** employee green behavior, self-esteem, perceived organizational support for employee environmental efforts, employee well-being

## Abstract

The issue of environmental protection and sustainable development is a key research focus across multiple fields. Employee green behavior is considered to be an important micro-activity to address this. Researchers in the field of organizational behavior and sustainable development have been focusing on the influencing factors of employee green behavior. However, few have explored the beneficial effects of employee green behavior on behavioral implementers. The objective of this study is to investigate the relationships among employee green behavior, self-esteem, perceived organizational support for employee environmental efforts, and employee well-being, and to explore a new dimension of employee green behavior. We empirically examined the underlying framework by conducting two surveys to collect data from 900 employees working in manufacturing, construction, and the service industry in China. We performed multilevel path analysis using SPSS and AMOS software, and confirmed that employee green behavior includes four dimensions: green learning, individual practice, influencing others, and organizational voices. Further, employee green behavior has a significant positive impact on self-esteem, which in turn is converted into employee well-being. Finally, perceived organizational support for employee environmental efforts not only positively moderated the relationship between employee green behavior and self-esteem, but was also confirmed as a moderated mediation model. This study enriches the current literature on the measurement framework and variables of employee green behavior.

## 1. Introduction

Research on the relationship between individual well-being and pro-social behavior has become an important well-being research topic [1,2]. Employee green behavior (EGB), a positive organizational behavior, is regarded as a micro-activity to solve environmental and sustainable development issues, which is essentially pro-social behavior. EGB refers to a series of behaviors implemented by employees in the workplace aimed at protecting the environment and promoting the sustainable development of the organization [3], including resource conservation, waste utilization, environmental protection knowledge acquisition, and sustainable work [4,5]. In the workplace, EGB can effectively promote organizational environmental performance and enable employees to have a greater sense of social gain [6,7].

Green behavior has become a common endeavor for companies and employees as a behavior to help both the organization and the environment [8,9]. Companies are encouraged by the pressure of environmental protection promotion to adopt green behavior as much as possible in specific work processes within the organization [10,11]. Many scholars have explored the factors and situations that affect the facilitation and manifestation of EGB [8,12], but few have explored the impact of EGB on the employees themselves [13,14]. Concerning existing research, at the organizational level, EGB is beneficial for reducing organizational resource consumption [15], and improves organizational environmental performance [16,17]. At the individual level, green behavior not only meets the requirements of work tasks or environmental protection goals, but also helps employees obtain task rewards, enhances job satisfaction [6,13], and has a positive impact on employees’ professional, physical, and mental health development [18].

Well-being is the common pursuit of both individuals and organizations. Individuals with a higher sense of well-being will have higher work motivation, and contribute more to productivity [19,20]. EGB, from the perspective of the implementer, and as a behavior helping both the organization and the environment, reflects the reciprocity in the sentiment of the old saying, “the roses in her hand, the flavor in mine.” When their material needs are met, employees are more concerned about self-realization and the improvement of well-being [21]. Zheng et al. [22] believe that employee well-being (EWB) is not only the employee’s cognition and perception of satisfaction at work and life, but also the emotional and psychological experience and the state of satisfaction manifested at both work and non-work levels. When employees implement green behavior and find that their behavior produces beneficial change, their sense of efficacy and meaning becomes satisfied, thereby promoting a greater sense of individual well-being. This fits the description of mutual benefit behavior in social exchange theory [23].

Previous studies also report that when employees feel the beneficial effects of implementing their behavior, they will make positive and subjective judgments on their own importance, ability, virtue, and strength—namely, self-esteem, satisfying their internal needs [6,24]. Therefore, self-esteem, as one relevant indicator for measuring well-being [25,26], may play an important role in the relationship between EGB and EWB. At the same time, when individuals self-judge their own behavior, they are also influenced by colleagues or organizations around them, and organizational support can deepen employees’ experience of well-being [27]. Perceived organizational support (POS) for employee environmental efforts—in other words, employees’ perception of whether their green behavior is recognized and valued by the organization—affects their attitudes and behavior [28]. Employees with a high POS for environmental efforts better understand the beneficial effects of implementing green behavior and can more easily improve their positive self-cognition and well-being. In the context that environmental concern has become the focus, more attention should be paid as to how pro-environmental behavior (green behavior) in the workplace can promote EWB.

This study focuses on the relationship between EGB and EWB and discusses how and when EGB affects EWB. Firstly, based on the procedural differences and internal logic generated by green behavior, four dimensions of EGB are proposed and verified: green learning, individual practice, influencing others, and organizational voices. Secondly, we construct a theoretical model of EGB, self-esteem, and EWB, and explore the path of influence of EGB, self-esteem, and POS for employee environmental efforts on EWB. We further investigate the mechanism between EGB and EWB and provide empirical support for the influence of pro-environmental behavior factors in the process of EWB in the workplace. This study enriches the dimensional research and the outcome variables of EGB, so that enterprises and employees can clearly realize the beneficial effects of green behavior on the implementers of behavior. The research conclusions enable managers to better understand EGB and provides them with theoretical guidance for promoting EGB and improving EWB.

## 2. Theoretical Basis and Research Hypothesis

### 2.1. Employee Green Behavior

Compared with the concept of individual green behavior, which refers to taking action that minimizes the negative impact on the environment or has a positive impact on environmental protection [29], EGB is environmentally friendly or pro-environmental behavior specific to the workplace [30,31]. It directly links environmental protection and other related positive actions with resource conservation in the daily operation of the enterprise, and helps organizations and employees to evaluate their own workplace green behavior [32]. Thus, it specifically determines the implementation effect of enterprises’ relevant green measures in the work process [33], and is the key to promoting the sustainable development of society and the economy [34].

The beneficial effects of EGB within organizations have aroused the interest of scholars in China and elsewhere. For example, experiments by Chen et al. [35] in the Chinese construction industry found that green behavior of employees decreases the generation of construction waste, thus reducing the consumption of organizational resources. Spontaneous green behavior such as employees’ active participation in environmental protection knowledge-sharing, identification of environmental issues, and suggestions to management are conducive to the improvement of organizational environmental performance [16]. In addition, empirical studies by Daily et al. [23], Paillé et al. [17], and Boiral et al. [36] have shown that various forms of green behavior such as employee environmental-oriented organizational citizenship behavior and the development of environmentally-friendly products are beneficial for improving the environmental performance of the organization.

While EGB improves the organization’s green performance, it also has an impact on an individual’s character traits. Research by Osbaldiston and Sheldon [37] has shown that EGB can promote the realization of goals, not only to meet the requirements of workplace tasks, but also to earn rewards and increase job satisfaction. Bauer and Aiman-Smith [18] proved that EGB can promote career development, and that individuals will also gain more satisfaction in the process of practicing green behavior [38]. Combing the existing research, we found that the antecedent variables of EGB are richer, focusing on individual factors such as emotion [11,39], self-efficacy [40], and job satisfaction [41], and contextual factors such as corporate social responsibility [42], leadership behavior [3], green atmosphere [43], and green human resource management practices [8,44], while the outcome variable research mainly focuses on three aspects—organizational green performance [16], employee job satisfaction [37], and career development [18].

Although studies have shown that EGB has a positive impact on both the organization and the individual, there is some controversy about the measurement framework of individuals’ green behavior within the enterprise. At present, there are many types of EGB scales, and most studies propose specific measurement frameworks based on two aspects—differences in actors and functional perspectives. Roberston et al. [45] developed a single-dimensional seven-item scale for EGB based on the definition of environmental behavior in the workplace by Ramus and Steger [30], combined with literature on environmental psychology. The seven-item scale specifically describes the individual environmental behavior of employees in the workplace, such as “I print double-sided whenever possible.” From the perspective of organizational voice behavior, Temminck et al. [28] used employee self-assessment to measure employees’ willingness in undertaking environmental protection behavior, and developed a single-dimensional seven-item scale. The measurement content is more abstract, such as “I try to draw management’s attention to potentially environmentally-unfriendly activities.” Erdogan et al. [46], also from the voice perspective, developed a single-dimensional five-item scale, which pays more attention to measuring employees’ voice behavior, such as “This employee helps come up with creative suggestions that have the potential to improve the organization’s environmental performance.”

There are also some measurement frameworks based on functional perspectives. Bissing-Olson et al. [32] developed a six-item daily task-related pro-environmental behavior scale and proactive pro-environmental behavior scale for employees by adapting Williams et al.’s [47] employee in-role performance scale and Frese et al.’s [48] personal initiative scale, such as “Today, I adequately completed assigned duties in environmentally-friendly ways.” and “Today, I did more for the environment at work than I was expected to.” Boiral and Paillé [49], based on the requirement type or in-role green behavior perspective, believe that EGB includes three dimensions—eco-initiatives, eco-civic engagement, and eco-helping—such as, respectively, “I voluntarily carry out environmental actions and initiatives in my daily work activities,” “I actively participate in environmental events organized in and/or by my company,” and “I encourage my colleagues to adopt more environmentally conscious behavior.” The above scales were developed from different perspectives: (1) based on differences among the actors, namely, two types of scales for individual practice and organizational voices; and (2) based on functional differences, developed through the in-role green behavior scale and the out-of-role green behavior scale, which can prompt colleagues into undertaking environmental protection behavior that is also a way for employees to implement green behavior.

We found that there is a certain gap between employees’ own ability and awareness when implementing green behavior. For individuals to effectively carry out green behavior in practice is a challenge. Therefore, it is particularly important to strengthen individual green learning. Moreover, most employees believe that green learning can not only strengthen their own green behavior ability, but also help raise awareness of green behavior implementation [50,51]. Green behavior implemented by employees in the workplace is not limited to one-sidedness. Individual practice, influencing others, and organizational voices are behavior that an employee can adopt in daily life. In addition, employees actively participating in green training and acquiring environmental protection knowledge in their daily work can raise awareness of environmental protection and resource conservation, and improve environmental cognition, enabling them to have a positive environmental attitudes and behavior [52,53]. Therefore, based on the learning, practice, encouragement of others, and positive influence on the implementation of the organization corresponding to the whole process of EGB, this paper proposes a four-dimensional measurement framework of EGB, including green learning, individual practice, influencing others, and organization voices. Taken together, we hypothesize that:

**Hypothesis 1.**  *EGB can be divided into the following four dimensions: green learning, individual practice, influencing others, and organizational voices.*

### 2.2. Employee Green Behavior and Employee Well-Being

“Well-being” focuses on the psychological feelings in the process of seeking happiness and avoiding pain. Its connotation is broader and deeper compared with the concept of “health” [54]. EWB refers to the positive mental health status of employees in the workplace, reflecting the individual physical arousal state and psychological satisfaction level at work, and is a measure of the mental health of organizational employees [55]. Studies have shown that individual feelings or experiences—such as moral values [56], psychological capital [57], inclusive leadership [58], and helping behavior [59]—can positively affect EWB. Pro-social behavior can improve individual subjective well-being, because altruistic behavior promotes more positive social interaction and integration among people, and can enhance an individual’s sense of life meaning [60]. Researchers are increasingly attaching importance to the significance of pro-social behavior to the parties concerned, and are comprehensively considering the self-improvement of the behavior implementers, the benefits of the behavior receivers, and the positive experience of both parties [61]. Pro-social behavior generally refers to all behavior that meets social expectations and is beneficial to others, groups, and society [62]. Green behavior is essentially behavior that helps organizations and the environment [63]. A large number of studies have found that pro-social behavior can bring happiness to the performer. For example, volunteering can make individuals better cope with psychological stress [64] and reduce depression symptoms [65]. Volunteers tend to have a higher level of mental health [66], and as such can experience greater happiness [67,68] and life satisfaction [69,70].

Well-being does not only refer to the feeling of realizing happiness. Whether an individual can achieve the perfect experience of the self-pursuit of goals by their own volition is the main criteria determining individuals’ well-being [71]. When the goals and behavior pursued by individuals are consistent with their values or interests, especially when the goals and behavior are spontaneously selected by them rather than forced externally, employees will obtain greater satisfaction and well-being [72]. Employees implement green behavior based on their own values and effort, without any external pressure, consistent with their goals and interests. Bauer and Aiman-Smith [18] showed that EGB is due to the pursuit of green and environmental protection, and this behavior bestows on the employees a greater sense of social gain, which not only enhances their own self-image, but also benefits their career, and physical and mental health. As a kind of pro-social behavior, EGB not only promotes the sustainable development of the organization and the environment, but also make the implementer feel meaningful and satisfied, thereby enhancing their well-being [2,73]. Therefore, EGB satisfies people’s need for finding the meaning of work and obtaining a rich life.

This study proposes that EGB can improve EWB based on the existing literature. As a spontaneous act of helping the organization and the environment, EGB enables the performer to respond to the green call and contribute to the sustainable development of the organization [16]. While gaining the trust and respect of the organization and its leadership, it also allows employees to feel their own sense of importance within the organization, increase positive emotions, and experience a greater sense of knowledge and intrinsic satisfaction arising from green behavior [18,38], thereby promoting EWB. On the contrary, if employees disregard the green development of the organization and squander organizational resources in the workplace, they will be criticized by their superiors, and will lose the trust and attention of colleagues and the organization, thereby reducing their positive psychological and emotional experience [74], reducing the EWB, and even producing negative emotions and attitudes [75]. In contrast, as a kind of pro-environmental and pro-organizational behavior, EGB can enable employees to actively and calmly cope with various pressures and challenges at work, thereby giving employees a greater sense of well-being. In summary, we propose the following hypothesis:

**Hypothesis 2.**  *EGB is positively associated with EWB.*

### 2.3. The Mediating Role of Self-Esteem

Leary and Baumeister [76] reported that self-esteem, as a stable personality trait, is an individual’s overall positive evaluation of the self. It reflects the individual’s continuous evaluation of their own abilities and value in the organization [77]. Coopersmith [78] believes that self-esteem mainly includes four aspects: importance, ability, virtue, and strength. Individuals with high self-esteem tend to view themselves from a positive perspective, believe in their multifaceted abilities, tend to change situations when faced with setbacks, and become more confident when faced with failure [79]. Therefore, self-esteem is considered to be one of the most reliable indicators of individual well-being [25,80]. Evidence suggests that self-esteem can mediate the effects of other variables on well-being [81].

Volunteer behavior can produce a sense of competence, participation, and usefulness [82]. When receiving the support and help provided by the giver, the beneficiary will have the obligation to repay and maintain the relationship between the two parties by implementing actions that are beneficial to the giver [83]. The behavior that employees implement in the workplace that are beneficial to the organization (EGB) is a very important factor affecting individual self-esteem. By helping others, organizations, or society, individuals can gain praise from others, which increases their self-esteem [84]. The research of Grube and Piliavin [82] shows that people who have long been engaged in voluntary activities usually gain greater self-understanding, which can significantly improve their self-esteem. The implementation of green behavior by employees not only responds to the call of a low-carbon society, but also benefits the development of the organization and the environment. They may also be recognized and supported by organizations and leaders in the workplace, forming a favorable green atmosphere [13]. Green behavior not only meets the requirements of work tasks or employees’ own environmental protection wishes, but the beneficial effects they bring can make employees perceive self-worth and improve self-esteem [6,85]. Therefore, EGB can satisfy psychological awareness of importance, abilities, virtues, and strengths, thereby enhancing employees’ self-esteem. Based on this, we formulate the following hypothesis:

**Hypothesis 3.**  *EGB is positively associated with self-esteem.*

Self-esteem is positively related to positive emotions [86]. When employees feel a greater level of self-esteem, their well-being will also increase, which fits self-determination theory, an important theoretical basis for well-being research [87]. The theory holds that, in essence, the individual is an active organism. Therefore, for well-being, the satisfaction of internal needs is more important than the satisfaction of external needs such as material goods and money [88]. The three basic psychological needs that determine well-being mainly include the need for autonomy, the need for competence, and the need for belonging [88]. The need for autonomy means that an individual must have a certain amount of control over their own behavior and be able to choose their own behavior according to their inner wishes. The need for competence means that an individual has the ability to complete a certain task to a satisfactory level and has a certain influence over the task, which are recognized by everyone in the organization. The need for belonging means that an individual wants to feel the care, respect and identification of others in the external environment, so as to experience a connection with the group [88]. Based on the existing literature, this study proposes that the construct of self-esteem can measure the psychological perception of employees from three aspects that affect EWB: the need for autonomy, the need for competence, and the need for belonging.

When employees have a high level of self-esteem, employees will think that their work is meaningful and believe that they are capable of completing certain tasks. At the same time, employees are also given a certain degree of autonomy in the process of completing tasks and can choose or control their own behavior or work style. In addition, employees also believe that they have an important influence on organizational strategy or management. These psychological perceptions all reflect that the basic inner needs of employees are met. According to self-determination theory, EWB will thus correspondingly increase [89,90,91]. On the contrary, if employees feel low self-esteem, they will consider that their work is not very meaningful, will have little confidence in their own abilities, have relatively low autonomy in the process of completing work tasks, and cannot have a significant influence on organizational strategy or decision-making. In such a case, the basic inner needs of employees are not met, and their level of well-being will be lower. Taken together, the following hypotheses are proposed:

**Hypothesis 4.**  *Self-esteem is positively associated with EWB.*

Inferred from Hypotheses 2–4.

**Hypothesis 5.**  *Self-esteem mediates the positive relationship between EGB and EWB.*

### 2.4. The Moderating Role of Perceived Organizational Support for Employee Environmental Efforts

POS refers to the degree to which employees perceive that the organization values their contributions and cares about their well-being [92], including perceived concern for employees’ interests, assistance provided to employees, and recognition of the value of employees’ work [93], which all impact employee attitudes and behavior [94]. Organizational support theory proposes that employees personalize the organization and may doubt the organization’s intentions for them [95], suggesting that individuals may have different perceptions of organizational support [96]. POS can meet individual social and emotional needs, such as praise, respect, sense of belonging, or emotional support [95], so POS can predict a series of work results such as engagement, task performance, organizational citizenship behavior, and EWB [97]. Organizational support will provide individuals with the necessary resources and support, and meet their social and psychological needs, which may affect the individual’s self-esteem. When employees perceive a higher level of organizational support for employee environmental efforts, they will better understand the beneficial effects of implementing green behavior, and will more easily obtain the resources and support they need, and more easily meet their social and psychological needs; their level of self-esteem will correspondingly improve. When employees have low POS for environmental efforts, they may ignore the beneficial effects of implementing green behavior, have difficulty in obtaining the required resources and support, are unable to meet their social and psychological needs, and their level of self-esteem may be low. Therefore, the following hypothesis is made:

**Hypothesis 6.**  *POS for employee environmental efforts positively moderates the relationship between EGB and self-esteem; that is, the higher the POS for employee environmental efforts, the stronger the positive impact of EGB on self-esteem, and vice versa.*

In this study, Hypotheses 2–5 indicate that self-esteem mediates the relationship between EGB and EWB, and Hypothesis 6 indicates that POS for employee environmental efforts plays a moderating role between EGB and self-esteem. Therefore, following the above logical hypothesis, we propose a regulated mediation model. Based on social exchange theory, employees who implement green behavior in the workplace are likely to increase their level of self-esteem, thereby enhancing their well-being. POS for employee environmental efforts, as a kind of employees’ perception of the organization’s importance to their implementation of green behavior, may play a moderating effect in the indirect role of self-esteem. As such, we develop the following hypothesis:

**Hypothesis 7.**  *POS for employee environmental efforts positively moderates the entire intermediary mechanism through which EGB affects EWB through self-esteem. That is, the higher the POS for employee environmental efforts, the stronger the mediating role of self-esteem, and vice versa.*

**Figure 1:**
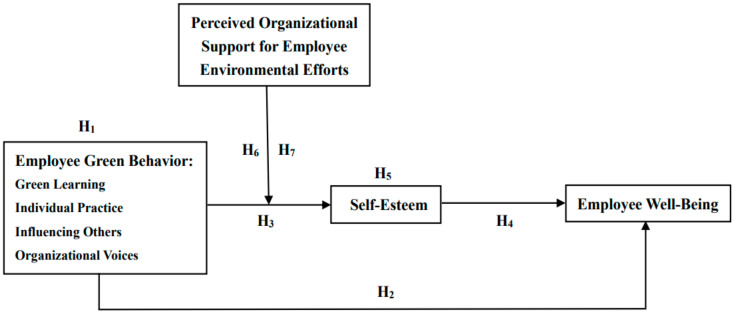
The hypothesized model.

In accordance with the literature review and the hypotheses outlined above, our research framework is illustrated in Figure 1.

## 3. Research Design on Employee Green Behavior

### 3.1. Pre-Investigation

Representative research studies on the green behavior of employees include surveys and studies on foreign employees’ green behavior by Robertson and Barling [45], Temminck et al. [28], and Boiral and Paillé [49]. Our research draws on their measurement scales, combined with China’s national conditions, literature research, and expert interviews, from which a preliminary questionnaire was constructed. The pre-survey was launched in September 2020, using anonymous fill-in methods to collect data from eight manufacturing companies in China. Among them, 101, 91, and 58 questionnaires were issued to three companies in Shandong, three companies in Anhui and two companies in Jiangsu, respectively, and 80, 67, and 39 questionnaires were effectively recovered. In total, 250 questionnaires were distributed, and 186 were well-completed.

### 3.2. Exploratory Factor Analysis

We carried out exploratory factor analysis (EFA) followed by reliability and validity tests of the 186 questionnaires. First, SPSS 25.0 was used to carry out the Kaiser–Meyer–Olkin (KMO) measure of sampling adequacy and Bartlett’s test of sphericity. We determined the KMO index to be 0.806 (>0.8), and Bartlett’s test of sphericity was statistically significant. Thus, the results were deemed suitable for EFA. Next, we followed the principles of the principal component extraction factor, the orthogonal variance maximal, and the Kaiser criteria (eigenvalue >1 rule), the factor load not being lower than 0.5 in EFA. Finally, the four-dimensional structure of the thirteen questions was obtained, explaining a total variance of 79.113%. The Cronbach alpha coefficient of each factor is above 0.8. Thus, the EFA results show that the green learning dimension in organizations exists in parallel with individual practice, influencing others, and organizational voice dimensions. In order to verify the EFA results (see Table 1) and conduct related hypothesis tests, we changed the survey subjects and expanded the sample data to do further empirical research.

## 4. Model Research Design

### 4.1. Sample and Procedure

Empirical data were collected from 650 employees from 23 medium-sized enterprises in China. The investigation work was conducted from August to October 2020. The companies that participated were mainly located in Shandong (7 companies, 196 data), Henan (6 companies, 179 data), Zhejiang (6 companies, 174 data), and Jiangsu (4 companies, 101 data), involved in manufacturing, construction, and the service industry. First of all, we identified the contact person of each company, explained the purpose, content, and process of the survey, and emphasized the anonymity of the questionnaire to obtain the trust of the contact person. Secondly, with the support of the human resource managers from the various companies, we sent a cover letter to each participant, which provided information about employee green behavior and the purpose of our research. Finally, in order to encourage the employees to complete the questionnaire seriously, we entrusted the human resources department to assist, so as to ensure accuracy of the responses and a high recovery rate of the questionnaire survey. In total, 448 valid questionnaires were received back.

### 4.2. Measures

The measures used in this study, except for the EGB scale, were adapted from existing validated scales. All were rated on a scale from 1 (strongly disagree) to 5 (strongly agree). 

Employee green behavior (EGB): The four sub-dimensions of EGB were measured using a scale developed in this study. Green learning was measured using three items, such as “I stay informed of my company’s environmental initiatives.” Individual practice was measured using four items, such as “I print double-sided whenever possible.” Influencing others was measured using three items, such as “I spontaneously give my time to help my colleagues take the environment into account in everything they do at work.” Organizational voices were measured using items such as “I make suggestions about environmentally-friendly practices to managers to increase the company’s environmental performance.” The Cronbach alpha coefficient was 0.909.

Self-esteem: We measured self-esteem using the Rosenberg ten-item scale [98], where there are four reverse measurement items, such as “All in all, I am inclined to feel that I am a failure.” The other six items are all positive measurements, such as “I feel that I am a person of worth, at least on an equal plane with others.” The Cronbach alpha coefficient was 0.938.

Perceived organizational support (POS) for employee environmental efforts: We selected the POS for employee environmental efforts questionnaire by Temminck et al. [28], which contains a total of seven items. The questionnaire also uses reverse and positive measurement standards, such as “The organization values my environmental contribution” and “The organization fails to appreciate any of my environmental efforts.” The Cronbach alpha coefficient was 0.915.

Employee well-being (EWB): EWB measures were adopted from Zheng et al.’s [22] 18-item scale. The scale contains three dimensions, namely, life well-being such as “Most aspects of my life are close to my ideals,” work well-being such as “My work is very interesting,” and psychological well-being such as “Generally speaking, I am positive about myself and full of confidence in myself.” It was developed in a Chinese organizational context and has been well-verified. The Cronbach alpha coefficient was 0.941.

Controlled variables: Previous studies have shown that variables such as employees’ gender, age, education and organizational tenure have a certain impact on employees’ attitudes and behavior [99]. Therefore, we controlled for the impact of these four variables on the main variables.

## 5. Data Results and Analysis

### 5.1. Model Testing

We first conducted a series of confirmatory factor analyses (CFA) using AMOS 25.0 software to examine the discriminant validity of the four sub-dimensions of EGB. We followed the Anderson and Gerbing two-step procedure for SEM analysis. First, a CFA model was specified and estimated [100]. This model included four sub-dimensions of EGB that were allowed the dimensions to correlate freely with one another. It met the criteria for a very good fit (χ^2^ = 115.736, df = 59, χ^2^/df = 1.962, IFI = 0.982, TLI = 0.976, CFI = 0.982, GFI = 0.962, RMSEA = 0.046) [101]. Furthermore, the fit of the four-factor model was superior to the fit of alternative models; thus, EGB’s four-dimensional structure was verified. The four-dimensional EGB could therefore be used for data collection, and its reasonable structure design could be used for hypothesis testing in this study. Hypothesis 1 was thus validated. The fit of each model is shown in Table 2.

Next, we tested the model as a mediating effect (three-factor measurement model), in which all indicators were loaded onto their respective latent variables (EGB, self-esteem, and EWB). An acceptable model fit was demonstrated (χ^2^ = 392.067, df = 109, χ^2^/df = 3.597, IFI = 0.940, TLI = 0.925, CFI = 0.940, GFI = 0.911, RMSEA = 0.076), with all standardized factor loadings significant at the *p* < 0.001 level. As such, we tested the mediation mechanism. On this basis, we added the moderation variable of POS for employee environmental efforts, and then carried out CFA on all four variables in the analysis. The results revealed a good fit to the data (χ^2^ = 688.743, df = 232, χ^2^/df = 2.969, IFI = 0.936, TLI = 0.923, CFI = 0.935, GFI = 0.892, RMSEA = 0.066). The four-factor model (EGB, self-esteem, POS for employee environmental efforts, and EWB) was, thus, retained for hypothesis testing.

### 5.2. Descriptive Statistics and Correlations

Table 3 presents the means, standard deviations, latent inter-correlations, and Cronbach alpha coefficients for the measured variables. All scales had acceptable internal reliabilities. These correlations show that EGB is positively correlated with EWB, and self-esteem and POS for employee environmental efforts also reveals a significant correlation with EWB.

### 5.3. Testing of Hypotheses

Main effect test: Using hierarchical regression analyses, we tested Hypothesis 2. Demographic controls including gender, age, education, and organizational tenure were entered first into all analyses. As displayed in Table 4, we determined that EGB had a direct, positive relationship on EWB (model 2, β = 0.270, *p* < 0.001). Therefore, Hypothesis 2 was verified. EGB positively affected self-esteem (model 6, β = 0.276, *p* < 0.001), thus supporting Hypothesis 3. In addition, as can be seen from Table 4, self-esteem had a direct, positive relationship on EWB (model 3, β = 0.359, *p* < 0.001), thus supporting Hypothesis 4.

The mediating effect test: After introducing the mediation variable (self-esteem) to the relationship between EGB and EWB, we found that the influencing co-efficient of EGB on the EWB (model 4, β = 0.184, *p* < 0.001) became smaller. However, the positive impact of self-esteem on EWB is still significant (model 4, β = 0.309, *p* < 0.001). The results of the analysis show that self-esteem played a mediating role between EGB and EWB. Thus, Hypothesis 5 was verified.

**Figure 2:**
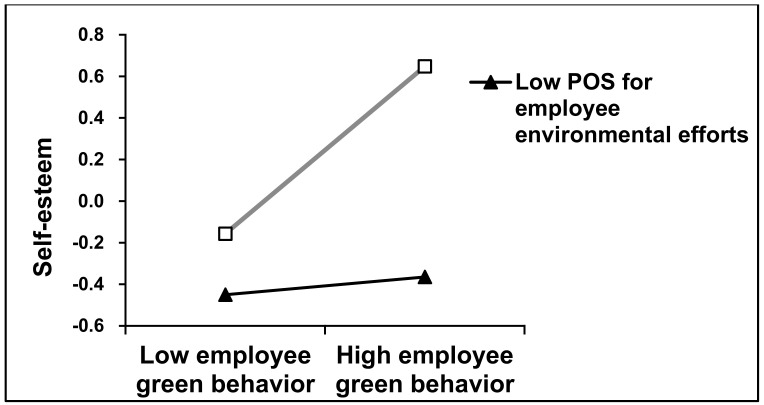
Moderating effect of perceived organizational support (POS) for employee environmental efforts.

The moderating effect of POS for employee environmental efforts: EGB and POS for employee environmental efforts both had a significant positive impact on self-esteem, respectively (Table 5). We further tested the hypothesized moderating effect of POS for employee environmental efforts on the relationship between EGB and self-esteem by entering the interaction terms between EGB and POS for employee environmental efforts. The results indicate that the interaction between EGB and POS for employee environmental efforts is significantly positively related to self-esteem (model 4, β = 0.222, *p* < 0.001), thus supporting Hypothesis 6. In order to more vividly present the moderating effect of POS for employee environmental efforts, we plotted interaction effects at different levels of POS for employee environmental efforts. As illustrated in Figure 2, the results reveal higher levels of POS for employee environmental efforts and higher levels of self-esteem, indicating a stronger positive relationship with regard to self-esteem. Therefore, Hypothesis 6 is again supported.

The moderated mediating effect test: We used the SPSS PROCESS plug-in, selecting a 95% confidence interval based on 5000 Bootstrap sampling tests, to obtain the results of the conditional indirect effect of POS for employee environmental efforts and the moderated mediating effect. Table 6 shows that the indirect effect value of self-esteem is 0.0142 (confidence interval [−0.0256, 0.0504]) under low POS for employee environmental efforts, and the indirect effect value of self-esteem is 0.1340 (confidence interval [0.0525, 0.2436]). With a high POS for employee environmental efforts, the confidence interval does not contain 0, and the moderated mediating effect is significant. In addition, when POS for employee environmental efforts was used as a moderating variable, the indirect effect value of self-esteem is 0.0874 (confidence interval [0.0266, 0.1715]), and the confidence interval does not contain 0, again verifying that the moderated mediating effect is significant. Thus, Hypothesis 7 is supported.

## 6. Discussion and Implications

This paper proposes a four-dimensional measurement framework of EGB, including green learning, individual practice, influencing others, and organization voices. The empirical results show that the reliability and discrimination validity of the four-dimensional structure combination are good. Previous studies on the measurement of EGB are mostly carried out from the aspects of individual practice, influencing others, and organizational voices [45,46,49]. It should be noted that how to identify and select green behavior is the key to employees’ implementation of green behavior [51]. Green learning behavior is the basis for individuals to implement green behavior scientifically [102]. Green learning behavior can not only reflect employees’ initiative in environmental protection, but also provide individual employees with motivational support for the implementation of daily work tasks as well as so-called subtle green behavior in the organization, including individual practice, influencing others, and organizational voices. This study is the first to use green learning behavior as an aspect of measuring EGB.

From the perspective of well-being, this study explored the outcome variables of EGB, constructed a theoretical model of EGB, self-esteem, and EWB, and empirically analyzed whether POS for employee environmental efforts plays a moderating role in the entire theoretical model. The results show that EGB is positively associated with EWB, supporting our research hypothesis. Self-esteem is positively associated with EWB, which is consistent with the findings of previous research [25,80], and self-esteem plays a part in mediating the relationship between EGB and EWB. POS for employee environmental efforts plays a moderating role in the positive relationship between EGB and self-esteem; that is, high POS for employee environmental efforts strengthens the positive impact of EGB on self-esteem. At the same time, POS for employee environmental efforts positively moderates the entire intermediary mechanism through which EGB affects EWB through self-esteem. That is, the higher the POS for employee environmental efforts, the stronger the mediating role of self-esteem. These research conclusions provide theoretical guidance for organizations to encourage employees to adopt green behavior and improve EWB.

### 6.1. Theoretical Implications

First, this study proposed a four-dimensional scale of EGB based on the initial acquirability of green behavior. Empirical analysis was carried out on this basis, and the results show that EGB can significantly promote EWB. EWB is the subjective feeling of employees’ satisfaction with life, work, and psychology [22]. Employees with higher well-being have better work performance, lower job burnout, and will more actively make environmentally friendly suggestions for the company. Therefore, it has an important impact on the survival and long-term development of the company [103]. The implementation of green behavior by employees in the workplace will inevitably cause a series of reactions in the organization, and EWB is a kind of emotional feeling in the process of interpersonal communication [104,105]. Consequently, from the perspective of EWB, it is of great significance to study EGB in the organizational context. Meanwhile, this research extends the focus of the existing literature by combining EGB with EWB, discovering a significant positive correlation between EGB and EWB. This result not only expands the research scope of the two aspects of pro-social behavior and EWB, and makes theoretical contributions, but also further promotes the development of positive organization behavior, promoting more scholars to pay attention to the importance of EGB as a research topic.

Second, the empirical results of this study show that self-esteem plays a mediating role between EGB and EWB. After clarifying the relationship between EGB and EWB, we further explore the internal mechanism and psychological process behind this relationship. Based on self-determination theory, this research proposes and reveals that self-esteem is an effective transmission mechanism that transfers the positive impact of EGB to EWB. In the early stage of the development of the theory of self-determination, scholars used the theory to predict EWB [88]. Later empirical studies used the theory to do related research on well-being [89]. However, Chinese research on self-determination theory is still mainly based on narrative research, while empirical research is relatively scarce [106]. Hence, this research makes an important contribution to the practical application of self-determination theory in the Chinese context. In addition, this study shows that EGB can improve employees’ self-esteem. This result not only expands the scope of the research on EGB, but also further expands the research on the antecedents of self-esteem.

Finally, the empirical results of this study show that POS for employee environmental efforts not only positively moderates the relationship between EGB and self-esteem, but also positively moderates the entire intermediary mechanism through which EGB affects EWB through self-esteem. We systematically analyzed the boundary conditions of EGB affecting self-esteem and found that this effect is more effective for employees with a higher sense of POS for employee environmental efforts. Moreover, when employees have a higher sense of POS for employee environmental efforts, the mediating role of self-esteem increases. This discovery expands the research on pro-social behavior and EWB.

### 6.2. Practical Implications

In order to promote the sustainable development of organizations and the pursuit of high well-being of employees, enterprises and individuals should take various measures to encourage the implementation of green behavior in the workplace. Drawing from the relevant research results of this article, we obtained the following insights for management: First of all, enterprises should pay more attention to green behavior of employees in human resource management practice (HRMP) [8,44]. Enterprises should not only pay consider their green values when recruiting employees, but also organize green training and learning to encourage employees to implement green behavior and improve their well-being. Secondly, in HRMP, enterprises have increased their attention on employee self-esteem. Thus, we recommend using specific methods to effectively identify employees with higher self-esteem levels, so as to harness this information and make it easier for employees to gain well-being in subsequent work tasks. In addition, employees should realize the importance of their green behavior to society, their organization, and themselves, strengthen their self-esteem, and promote the implementation of green behavior, which will in turn enhance their well-being. Finally, enterprises should carry out positive organizational behavior that demonstrates they care about employees in the daily management of their activities, and at the same time strengthen employees’ sense of organizational support [107,108], so that employees can perceive that their green behavior is recognized by organizations and leaders. As a consequence of promoting employees to adopt more green behavior, their well-being will be improved.

### 6.3. Limitations and Future Research

We explored the dimension of green learning through the study of the Chinese organizational context and proposed a four-dimensional measurement framework for EGB. The scale has been effectively validated in Chinese enterprises, but we are not sure whether it can be extended to other cultural contexts. Therefore, it is recommended that future research expand the scope of research and the research sample, and test whether it is applicable in the context of Western organizations. Secondly, all variables in this study are from the employee perspective. When employees filled out the questionnaire, the social approval effect may have come into play, which does not reflect the real situation. Follow-up research should improve the research design, conduct multi-source data collection, maximize the control of homology variance and data endogeneity, and more accurately verify the relationship between EGB and EWB. Finally, this study focuses on the overall concept of EWB. However, by definition, EWB comprises three dimensions: life well-being, work well-being, and psychological well-being [22]. In order to promote the in-depth development of EWB research, future research should further distinguish these three dimensions, and explore whether EGB has different degrees of impact on the three dimensions.

## 7. Conclusions

This study proposed that EGB also includes green learning, based on the initial acquirability of green behavior. Research has shown that EGB can be divided into four dimensions, including green learning, individual practice, influencing others, and organizational voices. In addition, the results suggest that EGB is an important predictor of EWB, and it is also implicated in the mediating effect of self-esteem and the moderating role of POS for employee environmental efforts. The results reveal that POS for employee environmental efforts not only positively moderates the relationship between EGB with self-esteem, but also positively moderates the entire intermediary mechanism through which EGB affects EWB through self-esteem. This study enriches the measurement framework and the outcome variables of EGB, so that enterprises and employees can clearly realize the beneficial effects of green behavior on the implementers of behavior. While promoting EWB, the research conclusions can better motivate employees to adopt green behavior and contribute to the sustainable development of the organization. Thus, policy makers should encourage employees to implement green behavior in the workplace, and give reasonable feedback on employees’ positive organizational behavior, so as to improve their self-esteem and well-being.

## Figures and Tables

**Table 1:** Four-dimensional exploratory factor analysis results of employee green behavior.

Questionnaire Item	Green Learning	Individual Practice	Influencing Others	Organizational Voices
1. I stay informed of my company’s environmental initiatives.	0.718			
2. I actively participate in environmental protection related training provided by the company.	0.901			
3. I take the initiative to learn environmental protection knowledge to improve environmental protection capabilities.	0.880			
4. I print double-sided whenever possible.		0.785		
5. I use personal water cups instead of disposable paper cups in the office.		0.701		
6. I complete the tasks assigned by the company in an environmentally friendly way.		0.898		
7. I perform the duties specified in the job description in an environmentally friendly manner.		0.821		
8. I spontaneously give my time to help my colleagues take the environment into account in everything they do at work.			0.893	
9. I encourage my colleagues to adopt more environmentally conscious behavior.			0.900	
10. I encourage my colleagues to express their ideas and opinions on environmental issues.			0.901	
11. I make suggestions about environmentally friendly practices to managers to increase company’s environmental performance.				0.849
12. I try to draw management’s attention to potentially environmentally unfriendly activities.				0.857
13. I inform management of potentially environmentally irresponsible policies and practices.				0.856
Interpretable variation (%) (79.113% overall)	18.319	21.487	20.664	18.644
Cronbach alpha coefficient (total 0.852)	0.848	0.840	0.932	0.878

**Table 2:** Confirmatory factor analysis results of the four-dimensional model for employee green behavior.

Models	χ^2^	DF	χ^2^/df	RMSEA	GFI	CFI	TLI	IFI
Single-factor model	689.879	65	10.614	0.147	0.781	0.804	0.765	0.805
Two-factor model	369.653	62	5.962	0.105	0.907	0.904	0.879	0.904
Three-factor model	564.032	64	8.813	0.132	0.841	0.843	0.809	0.844
Four-factor model	115.736	59	1.962	0.046	0.962	0.982	0.976	0.982

Notes: N = 448. Single-factor model: all four dimensions (green learning, individual practice, influencing others, and organizational voices) were loaded on the same factor. Two-factor model: individual practice, influencing others, and organizational voices were loaded on the same factor. Three-factor model: individual practice and influencing others were loaded on the same factor. Four-factor model: each of the four dimensions was loaded on an independent factor.

**Table 3:** Descriptive statistics and inter-correlations.

Variable	M	SD	1	2	3	4	5	6	7	8
1. Gender	1.527	0.500								
2. Age	1.580	0.677	−0.218 **							
3. Education	1.911	0.592	0.008	0.107 *						
4. Organizational tenure	2.616	1.405	−0.272 **	0.780	0.152 **					
5. Employee green behavior	4.206	0.560	0.006	0.022	−0.187 **	−0.026	(0.909)			
6. Self-esteem	4.144	0.575	0.044	−0.067	−0.138 **	−0.004	0.284 **	(0.938)		
7. POS for employee environmental efforts	3.933	0.685	0.025	−0.013	−0.160 **	−0.027	0.452 **	0.370 **	(0.915)	
8. Employee well-being	3.843	0.593	−0.015	0.051	−0.245 **	0.017	0.309 **	0.377 **	0.445 **	(0.941)

Notes: * *p* < 0.05. ** *p* < 0.01; N = 448; Scale reliabilities (Coefficient alpha) are on the diagonal. All the scales range from 1 to 5.

**Table 4:** Mediating role of self-esteem.

Variable Type		Employee Well-Being				Self-Esteem	
		Model 1	Model 2	Model 3	Model 4	Model 5	Model 6
Control variable	Gender	0.003	0.001	−0.016	−0.015	0.053	0.050
	Age	0.089	0.061	0.149 *	0.122	−0.167 *	−0.195 **
	Education	−0.253 ***	−0.202 ***	−0.201 ***	−0.173 ***	−0.145 **	−0.094 *
	Organizational tenure	−0.013	0.007	−0.071	−0.049	0.162 *	0.183 *
Independent variable	Employee green behavior		0.270 ***		0.184 ***		0.276 ***
Mediating variable	Self-esteem			0.359 ***	0.309 ***		
F		7.851	13.919	20.886	20.894	3.748	10.446
R^2^		0.066 ***	0.136 ***	0.191 ***	0.221 ***	0.033 *	0.106 ***
ΔR^2^		0.008	0.010	0.009	0.010	0.009	0.010

Notes: * *p* < 0.05. ** *p* < 0.01. *** *p* < 0.001; N = 448.

**Table 5:** Moderating role of perceived organizational support (POS) for employee environmental efforts.

Variable Type		Self-Esteem				Employee Well-Being	
		Model 1	Model 2	Model 3	Model 4	Model 5	Model 6
Control variable	Gender	0.053	0.050	0.043	0.024	−0.008	−0.016
	Age	−0.167 *	−0.195 **	−0.173 *	−0.190 **	0.082	0.070
	Education	−0.145 **	−0.094 *	−0.087	−0.072	−0.186 ***	−0.074 ***
	Organizational tenure	0.162 *	0.183 *	0.165 *	0.168 *	−0.009	−0.005
Independent variable	Employee green behavior		0.276 ***		0.222 ***		0.140 **
Moderator	POS for employee environmental efforts			0.358 ***	0.317 ***	0.417 ***	0.380 ***
Interactive item	Employee green behavior POS for employee environmental efforts				0.222 ***		0.095 *
F		3.748	10.446	16.492	17.235	27.1754	21.139
R^2^		0.033 **	0.106 ***	0.157 ***	0.215 ***	0.235 ***	0.252 ***
ΔR^2^		0.009	0.010	0.009	0.012	0.009	0.012

Notes: * *p* < 0.05. ** *p* < 0.01. *** *p* < 0.001; N = 448.

**Table 6:** The mediating effect and its 95% confidence interval at different levels of moderation.

Moderator	Employee Green Behavior Self-Esteem Employee Well-Being							
POS for employee environmental efforts	Conditional indirect effect				Moderated mediating effect			
	Effect	Standard error	Upper limit	Lower limit	Effect	Standard error	Upper limit	Lower limit
Low POS	0.0142	0.0190	−0.0256	0.0504				
High POS	0.1340	0.0484	0.0525	0.2436	0.0874	0.0368	0.0266	0.1715

Notes: POS, perceived organizational support.

## Data Availability

The raw data supporting the conclusions of this article will be made available by the authors, without undue reservation, to any qualified researchers.
